# Standardized ileal digestible lysine requirement of pregnant sows under commercial conditions

**DOI:** 10.5713/ab.23.0260

**Published:** 2023-10-20

**Authors:** Hyunwoong Jo, Beob Gyun Kim

**Affiliations:** 1 Department of Animal Science and Technology, Konkuk University, Seoul 05029, Korea

**Keywords:** Gestating Sows, Lysine, Requirements, Standardized Ileal Digestibility, Swine

## Abstract

**Objective:**

The present experiment aimed to determine standardized ileal digestible (SID) lysine (Lys) requirements for pregnant sows individually housed under commercial farm conditions.

**Methods:**

Two hundred multiparous sows (parity = 5.1±2.0) on day 42 of gestation were randomly allocated to five dietary treatments with a balanced parity. Experimental diets were formulated to contain 0.22%, 0.32%, 0.42%, 0.52%, and 0.62% of SID Lys for the mid-gestation period (days 42 to 76) and 0.36%, 0.46%, 0.56%, 0.66%, and 0.76% of SID Lys for the late gestation period (days 77 to 103). All indispensable amino acids except Lys were provided at 110% of their requirement estimates. Daily feed allowance per sow was determined based on the back-fat thickness and body condition score at the second pregnancy check and on day 90 of gestation. Three different statistical models were used to estimate the SID Lys requirement.

**Results:**

Total born piglets alive per litter increased linearly and quadratically (p<0.001) as dietary SID Lys increased. For total born piglets alive per litter, the SID Lys requirement estimates ranged from 9.69 to 12.4 g/d for the mid-gestation period (1.19 to 1.52 g/Mcal metabolizable energy; 0.39% to 0.49%) and 14.6 to 17.4 g/d for the late gestation period (1.62 to 1.93 g/Mcal metabolizable energy; 0.52% to 0.62%).

**Conclusion:**

The mean values of the SID Lys requirement for the mid-gestation period and the late gestation period are 11.1 and 16.1 g/d (1.36 and 1.79 g/Mcal metabolizable energy; 0.44% and 0.58%), respectively, for maximal total born piglets alive per litter.

## INTRODUCTION

Over the past 30 years, proliferation, leanness, and weight of modern sows have considerably changed [1]. These changes suggest that the nutrient requirements of gestating sows may have increased, and thus, it is necessary to re-evaluate these requirements. Moreover, gestating sows’ amino acid (AA) requirements considerably change as gestation progresses. This is due to the recovery of maternal tissues, fetal growth, and the development of mammary glands [2–4]. Therefore, estimating the optimal AA requirement for gestating sows as gestation progresses is important to ensure optimal reproductive performance.

In corn-soybean meal-based swine diets, lysine (Lys) is generally the first limiting AA, which has been used as a standard for other indispensable AA requirements [5,6]. Stein et al [7] proposed that standardized ileal digestible (SID) Lys is a more appropriate measure for expressing the bioavailability of Lys in ingredients and diets. Based on these concepts, the NRC [6] recommended that Lys requirement estimates should be expressed on an SID basis and employed a modeling approach to estimate AA requirements for gestating sows. However, the data for standardized ileal digestibility of gestating sows in the AA requirement model were derived from growing pigs. Furthermore, the NRC [6] relied on only four empirical studies [8–11] to model the Lys requirements. Therefore, an empirical approach is necessary to validate the model for gestating sows by determining their Lys requirements. Therefore, the objective of the present study was to determine the SID Lys requirement for gestating sows at different stages of gestation based on their reproductive performance under commercial conditions.

## MATERIALS AND METHODS

The experimental protocol was approved by the Institutional Animal Care and Use Committee of Konkuk University (KU15128).

The purpose of this study was to investigate the effects of varying dietary Lys concentrations on the reproductive performance of gestating sows during two stages of gestation: mid-gestation (MG; days 42 to 76) and late gestation (LG; days 77 to 103). This study utilized five different experimental diets administered to pregnant sows at various stages of gestation.

### Animals, diets, and experimental design

After three weeks of intrauterine insemination, the sows were diagnosed as pregnant by ultrasound, and a second confirmation was conducted three weeks later. A total of 200 multiparous gestating sows (Yorkshire×Landrace, Darby Queen-S; DARBY Co., Ltd, Anseong, Korea) on day 42 of pregnancy were used in this study.

All experimental diets were formulated on a SID basis using SID AA concentrations for each ingredient provided by the NRC [6]. To achieve 0.22% and 0.36% SID Lys in MG and LG, respectively, two low-Lys diets were used (Table 1), which represented 57% and 64% of the SID Lys requirements presented in the literature [3,6,11,12]. To obtain 0.32%, 0.42%, 0.52%, and 0.62% SID Lys diets in MG, four additional diets were prepared by replacing monosodium glutamate with crystalline Lys. Similarly, for LG diets, various SID Lys concentrations (0.36%, 0.46%, 0.56%, 0.66%, and 0.76%) were prepared using the same method as for MG diets. All experimental diets contained similar levels of crude protein and metabolizable energy (ME) among the treatments, and all indispensable AA, except Lys, were provided at 110% of their requirement estimates [6] for each gestation phase. The analyzed AA concentrations in the experimental diets are presented in Table 2. In addition, vitamins and minerals were included in all diets to meet or exceed the nutrient requirement estimates [6]. The gestating sows were randomly assigned to five dietary treatments with balanced parity (average parity = 5.1±2.0).

### Feeding, housing, and measurements

Gestating sows were individually housed in gestation crates. The amount of feed allowance per sow was determined based on the recommended feed ration for commercial farms, which was adjusted according to the back fat thickness and body condition score at the second pregnancy check on day 40 of gestation. The feed allowance was increased by 20% on day 90 of gestation until farrowing and the sows were fed twice a day at 0700 and 1630 h. Water was provided to the sows using a nipple drinker and was always available. The sows were fed the MG diets for 35 d (from gestation days 42 to 76) and then switched to the LG diets until they were transferred to the farrowing crate. After parturition, all the sows were fed a commercial diet. The total born piglets per litter (including live and stillborn piglets) was recorded for each sow, and the weight of each piglet, including the stillborn piglets, was recorded 24 h after birth. Litter mortality rate at birth was calculated based on total born piglets per litter and the number of stillbirth and dead piglets within 24 h after birth. Cross-fostering of piglets was carried out within each treatment and conducted within 72 h after their birth. The number of suckling piglets per sow was maintained similarly. After cross-fostering, the weight of each piglet was recorded, and lactation performance was evaluated by measuring the number of fostering piglets per litter for lactation, litter weight gain, and the number of piglets at weaning per litter. Additionally, the reproductive performance of each sow at the subsequent parity was recorded.

### Chemical analysis

The diets were finely ground before chemical analysis [13]. The experimental diets were analyzed for crude protein (method 990.03), calcium (method 978.02), phosphorus (method 964.06), and AA concentrations (method 982.30). Methionine was analyzed as methionine sulfone after cold performic acid oxidation overnight prior to hydrolysis. Tryptophan was analyzed following 4 *N* NaOH hydrolysis for 22 h at 110°C.

### Statistical analysis

Daily Lys intake was used as an independent variable to determine the SID Lys requirement and was expressed as grams per day rather than as a percentage because daily feed intake varied among the sows. Reproductive performance data were analyzed by analysis of variance using the general linear model procedure of SAS 9.4 (SAS Inst. Inc., Cary, NC, USA). The statistical model included daily Lys intake (g/d) as an independent variable. Linear and quadratic effects of increasing daily Lys intake were analyzed using orthogonal polynomial contrasts. Least squares means were calculated for each dependent variable and the experimental unit was a sow. Least squares means for the reproductive performance data of subsequent parity were compared using the PDIFF option with Tukey’s adjustment. When quadratic responses (p<0.05) were detected in the experimental data, the optimal SID Lys requirements were estimated using a broken-line analysis with the NLIN procedure of SAS. For all analyses, p<0.05 and 0.05≤p<0.10 were considered as statistical significance and tendency, respectively.

## RESULTS

During the experimental period, 14 gestating sows were excluded from the dataset due to miscarriages, foot disorders, or sudden death.

### Farrowing performance

The farrowing performance was measured before cross-fostering (Table 3). As the dietary SID Lys concentration increased, total born piglets per litter tended to linearly increase (p = 0.089). While litter weight at birth was not affected by dietary SID Lys concentration, the variation in litter weight at birth showed a quadratic tendency (p = 0.066) with increasing SID Lys concentration. Additionally, the litter mortality rate at birth quadratically decreased (p = 0.020) with increasing SID Lys concentration. Subsequently, total born piglets alive per litter increased linearly and quadratically (p<0.001) as dietary SID Lys increased.

**Figure 1:**
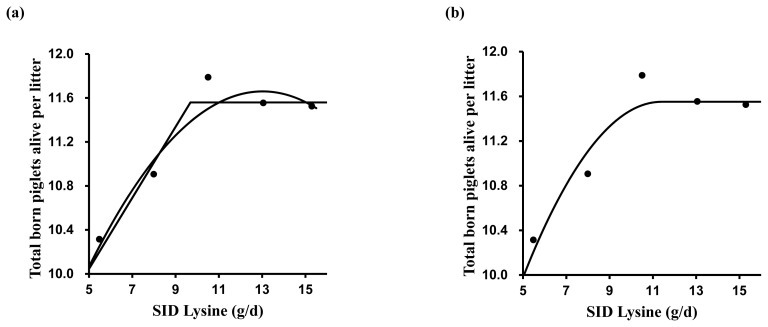
The broken-line analysis of total born piglets alive per litter in response to increasing standardized ileal digestible (SID) lysine (Lys) intake during mid-gestation (days 42 to 76) in pregnant sow. Data points represent least squares means of five dietary treatments in mid-gestation. Each regression model shows total born piglets alive per litter relative to dietary SID Lys intake per day. (a) One slope broken-line model indicated that the SID Lys requirement was 9.7 g/d (standard error [SE] = 1.08) based on the following equation: Y = 11.56–0.32×(9.7–X) where X is less than 9.7, with p<0.001. Quadratic model indicated that the SID Lys requirement was 12.4 g/d (SE = 0.91), which value was obtained from 95% of the upper asymptotic value of the model: Y = 11.7–0.02×(13.0–X)^2^, with p<0.01. The intercept between the two models indicated that the SID Lys requirement was 11.0 g/d. (b) Quadratic broken-line model indicated that the SID Lys requirement was 11.4 g/d (SE = 1.89) based on the following equation: Y = 11.6–0.04×(11.4–X)^2^ where X is less than 11.4, with p<0.01.

**Figure 2:**
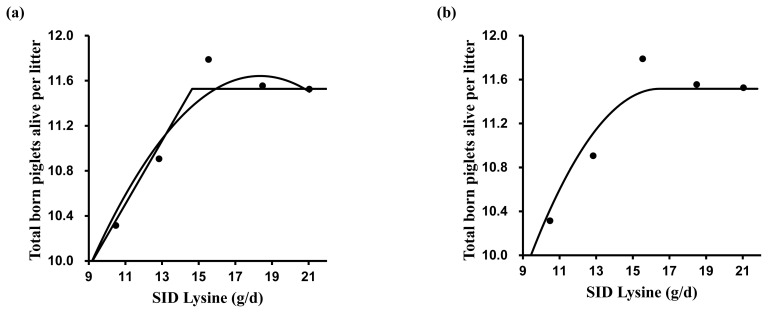
The broken-line analysis of total born piglets alive per litter in response to increasing standardized ileal digestible (SID) lysine (Lys) intake during late gestation (days 77 to 103) in pregnant sow. Data points represent least squares means of five dietary treatments in late gestation. Each regression model shows total born piglets alive per litter relative to dietary SID Lys intake per day. (a) One slope broken-line model indicated that the SID Lys requirement was 14.6 g/d (standard error [SE] = 1.16) based on the following equation: Y = 11.5–0.28×(14.6–X) where X is less than 14.6, with p<0.001. Quadratic model indicated that the SID Lys requirement was 17.4 g/d (SE = 0.86), which value was obtained from 95% of the upper asymptotic value of the model: Y = 11.6–0.02×(18.4–X)^2^, with p<0.01. The intercept between the two models indicated that the SID Lys requirement was 16.2 g/d. (b) Quadratic broken-line model indicated that the SID Lys requirement was 16.5 g/d (SE = 2.19) based on the following equation: Y = 11.5–0.03×(16.5–X)^2^ where X is less than 16.5, with p<0.01.

A broken-line analysis was conducted to determine the optimal dietary SID Lys for reproductive performance (Table 4). Various models were tested for estimating SID Lys requirements. The one slope broken-line analysis (2 straight-line, one-breakpoint model) showed that the optimal SID Lys requirement in MG and LG was 9.7 and 14.6 g/d, respectively (Figures 1a and 2a). The SID Lys requirement in MG and LG based on the former intercept value between the quadratic model and the plateau line of one slope broken-line model was 11.0 and 16.2 g/d (Figure 1a and 2a). According to the quadratic broken-line analysis for SID Lys requirement, the optimal SID Lys requirement in MG and LG was 11.4 and 16.5 g/d (Figure 1b and Figure 2b). Based on the average daily feed intake and ME concentration in diets, the requirements were converted to 1.19, 1.52, 1.35, and 1.40 g Lys/Mcal ME in MG and 1.62, 1.93, 1.80, and 1.83 g Lys/Mcal ME in LG.

### Lactation performance

Lactation performance data from 4 sows (1, 1, and 2 sows from the first, the third and the fifth treatment group, respectively) were not included in the calculations. These sows did not secret enough milk to foster the offspring nor were not able to stand. The number of fostering piglets per litter exhibited an increase (linear and quadratic p<0.001) with increasing dietary SID Lys concentrations (Table 5). Litter weight at weaning tended to linearly increase (p = 0.051) with increasing dietary SID Lys concentrations. Additionally, the litter weight gain linearly increased (p = 0.050) with increasing dietary SID Lys concentrations. Although the litter mortality rate at weaning linearly increased (p = 0.023), the number of piglets per sow at weaning also linearly increased (p = 0.026) with increasing SID Lys concentrations.

### Sow performance during the subsequent parity

After the experiment period concluded, the sows were fed a commercial diet during one reproductive cycle. No difference was observed in weaning to estrus interval or total born piglets per litter among dietary treatments (Table 6). However, the number of piglets at weaning differed (p = 0.047) among the treatment groups.

## DISCUSSION

The NRC [14] suggested a single value of AA requirement throughout the gestation period. However, this approach has been challenged by modeling studies [15–17] and empirical experiments [18,19], which have proposed changing requirements as gestation progresses. McPherson et al [20] and Ji et al [21] reported a marked increase in fetal growth during the last 45 days of gestation. Based on these findings, the NRC [6] suggested dividing the AA requirements of gestating sows into two phases. Samuel et al [3] confirmed that the requirement for gestating sows increased substantially from early gestation (days 24 to 45) to late gestation (day 86 to 110). Consequently, the experimental period in the present work was divided into MG (days 42 to 76 of gestation) and LG (days 77 to 103 of gestation) periods.

The AA requirements for gestating sows are influenced by several factors including protein gain in different protein pools, the efficiency of using SID AA intake for these functions, basal endogenous losses of AA from the gastrointestinal tract, and integument losses [6]. Additionally, the parity of sows can also affect the AA requirements due to physiological changes that occur with increasing parity [17]. To account for this, the parity of the gestating sows was balanced per treatment in the present study. Moreover, on day 90 of gestation, individual feed allowance was increased to prevent energy deficiency during the LG period.

Although increasing dietary SID Lys tended to increase total born piglets per litter containing stillbirths in the present study, no effects of dietary Lys on the number of total born size were reported in multiple experiments [22–24]. This discrepancy is likely due to the very low Lys concentrations (0.22%) in the lowest AA group at MG in the present study. Inadequate provision of dietary Lys to gestating sows can result in fetal death, leading to stillbirths with mummies, which typically occur between day 60 and 100 of gestation, resulting in smaller total born piglets with a large number of mummies [25–27]. In the present study, accordingly, the litter mortality rate at birth quadratically decreased and total born piglets alive per litter quadratically increased with increasing dietary SID Lys, which agrees with Seoane et al [28] who found that multiparous sows consuming 10.0 g SID Lys/kg during the LG period (days 77 to 107) had a higher litter born alive compared to those consuming 6.0 g SID Lys/kg.

While many studies have reported that low dietary Lys intake results in low birth weight [29–31], the present study did not find a significant effect of dietary SID Lys intake on litter weight at birth. One possible reason for this is that increasing dietary SID Lys tends to increase total born piglets per litter, which may lead to a restricted nutrient supply per fetus in utero. This finding is consistent with previous studies [32,33] and partially explains why higher dietary SID Lys intake did not influence litter weight at birth in this study.

The linear effects of dietary SID Lys intake on litter weight gain, litter weight, and number of piglets at weaning per litter in the present study are consistent with the results in previous studies [29,34,35]. Although the litter mortality rate at weaning increased linearly, the number of fostering piglets per litter also increased most likely due to the increased total born piglets alive per litter with increasing dietary SID Lys intake. There are several possible reasons for the improved lactation performance of sows with increased Lys intake during gestation. One reason for this is that feeding sows a high level of Lys during gestation may allow them to accumulate sufficient protein and develop mammary glands [4], which contributes to milk production for nutrient provision [30]. Another reason is that inadequate Lys intake during gestation could damage fetuses which could affect their postnatal performance [36].

Many previous studies have examined the impact of nutrition on sow longevity, but the results have been inconsistent. Sows fed the AA-unbalanced gestation diet had more maternal fat but less protein accumulation than those fed the AA-balanced diet, leading to greater mobilization in sows fed the AA-unbalanced diet [37]. Thus, these sows may have poor body conditions and decreased lactation ability [30,35].

Statistical methodology is one of the factors that affect the optimal requirement of nutrients of interest [38]. The use of different statistical models can lead to variations in the requirement values; thus, multiple methods have been used to prevent bias. The average values for SID Lys requirement based on different approaches were 11.1 and 16.2 g/d in MG and LG, respectively. According to 2.5 and 2.8 kg/d feed intake, the SID Lys requirements were converted to 0.44% and 0.58% in each gestation period. The increase in fetal growth and protein gain with advancing gestation is one of the reasons for the difference in requirements, as supported by empirical studies [3,24,27]. Samuel et al [3] estimated the SID Lys requirement during early gestation (days 24 to 45) and LG (days 86 to 110) using the indicator AA oxidation method. The six test diets provided SID Lys intakes of 7.5 to 19.3 g/d in early gestation and 8.1 to 23.7 g/d in LG. The authors reported that SID Lys requirements for early and late gestations were 9.4 and 17.4 g/d, respectively. Shi et al [24] fed five concentrations of dietary SID Lys (8.6 to 16.0 g/d) from day 1 to 80 of gestation then increased feed intake to provide 12.9 to 24.0 g/d SID Lys from day 80 to 110 of gestation. Lastly, the authors reported a decrease in the birth weight coefficient of variation when SID lysine was increased to 14.0 and 21.0 g/d before and after day 80 of gestation, respectively. Thomas et al [27] provided SID Lys (11.0 to 18.5 g/d) from day 5 to 111 of gestation and reported that the percentage of piglets born alive increased with increasing SID Lys intake, as a result of a reduction in the proportion of stillborn pigs.

In conclusion, the SID Lys requirements in MG and LG for optimal litter born alive of gestating sows were 0.44% and 0.58% (1.36 g/Mcal ME; 1.79 g/Mcal ME), which suggests that the different diets should be provided to the sows to satisfy the Lys requirements throughout gestation.

## Figures and Tables

**Table 1:** Ingredient and chemical composition of experimental diets (as-fed basis)

Item (%)	Low-lysine diet	

	Mid-gestation	Late gestation
Ingredient		
Ground corn, yellow dent	83.2	77.4
Corn gluten meal	6.00	3.80
Wheat bran	5.00	9.00
Soybean meal, 48% crude protein	-	4.00
Soybean oil	1.00	1.00
Monosodium glutamate	1.50	0.51
L-Lys•HCl, 78.8%	-	0.07
DL-Met, 99%	-	0.02
L-Thr, 99%	0.08	0.15
L-Trp, 99%	0.03	0.04
Monocalcium phosphate	1.00	1.35
Limestone, ground	1.15	1.70
Vitamin-mineral premix^1)^	0.50	0.50
Sodium chloride	0.50	0.50
Analyzed composition		
Crude protein	11.3	11.8
Calcium	0.86	1.30
Phosphorus	0.49	0.65
Lysine	0.33	0.39
Calculated composition		
Standardized ileal digestible lysine	0.22	0.36
Crude protein	11.9	12.2
Metabolizable energy (kcal/kg)	3,257	3,210

1) Provided the following quantities per kg of complete diet: vitamin A, 25,000 IU; vitamin D_3_, 4,000 IU; vitamin E, 50 IU; vitamin K, 5.0 mg; thiamin, 4.9 mg; riboflavin, 10.0 mg; pyridoxine, 4.9 mg; vitamin B_12_, 0.06 mg; pantothenic acid, 37.5 mg; folic acid, 1.10 mg; niacin, 62 mg; biotin, 0.06 mg; Cu, 25 mg as copper sulfate; Fe, 268 mg as iron sulfate; I, 5.0 mg as potassium iodate; Mn, 125 mg as manganese sulfate; Se, 0.38 mg as sodium selenite; Zn, 313 mg as zinc oxide; and butylated hydroxytoluene, 50 mg.

**Table 2:** Analyzed amino acids concentration in experimental diets (as-fed basis)^1)^

Item (%)	Standardized ileal digestible lysine (%)									

	Mid-gestation					Late gestation				
	
	0.22	0.32	0.42	0.52	0.62	0.36	0.46	0.56	0.66	0.76
Indispensable amino acids										
Arg	0.44	0.43	0.46	0.45	0.45	0.46	0.54	0.55	0.53	0.55
His	0.26	0.24	0.26	0.26	0.25	0.26	0.28	0.28	0.27	0.28
Ile	0.33	0.31	0.34	0.32	0.32	0.32	0.34	0.35	0.33	0.35
Leu	1.44	1.41	1.49	1.35	1.42	1.26	1.26	1.29	1.24	1.24
Lys	0.33	0.38	0.55	0.63	0.70	0.39	0.69	0.74	0.83	1.10
Met	0.27	0.28	0.28	0.30	0.27	0.29	0.27	0.26	0.25	0.26
Phe	0.28	0.41	0.38	0.34	0.42	0.33	0.37	0.40	0.37	0.36
Thr	0.47	0.48	0.55	0.49	0.46	0.51	0.57	0.56	0.56	0.55
Trp	0.07	0.06	0.08	0.07	0.07	0.10	0.10	0.14	0.08	0.11
Val	0.40	0.41	0.45	0.43	0.45	0.42	0.43	0.44	0.44	0.45
Dispensable amino acids										
Ala	0.82	0.81	0.86	0.79	0.81	0.72	0.74	0.75	0.74	0.73
Asp	0.74	0.72	0.76	0.73	0.71	0.77	0.85	0.87	0.83	0.87
Cys	0.32	0.33	0.35	0.35	0.32	0.35	0.35	0.35	0.32	0.33
Glu	3.23	3.33	3.36	3.15	2.77	2.40	2.27	2.40	2.21	2.13
Gly	0.40	0.39	0.42	0.40	0.38	0.39	0.44	0.43	0.44	0.44
Pro	0.82	0.81	0.84	0.78	0.80	0.73	0.76	0.76	0.75	0.76
Ser	0.58	0.57	0.61	0.57	0.57	0.55	0.59	0.60	0.58	0.59
Tyr	0.59	0.57	0.62	0.57	0.57	0.55	0.58	0.59	0.57	0.58

1) Mid-gestation, days 42 to 76; late gestation, days 77 to 103.

**Table 3:** Effects of standardized ileal digestible (SID) lysine (Lys) concentrations on the farrowing performance^1)^

Items	Standardized ileal digestible lysine (%)										SEM	p-value	
	
	MG	LG	MG	LG	MG	LG	MG	LG	MG	LG		Linear	Quadratic
				
	0.22	0.36	0.32	0.46	0.42	0.56	0.52	0.66	0.62	0.76			
Average daily feed intake (kg/d)	2.50	2.80	2.51	2.79	2.51	2.78	2.52	2.80	2.48	2.77	-	-	-
SID Lys intake (g/d)	5.48	10.5	8.00	12.8	10.5	15.5	13.0	18.5	15.3	21.0	-	-	-
Average parity	5.08		5.08		5.08		5.08		5.05		-	-	-
Number of farrowing sow	38		37		34		37		40		-	-	-
Total born piglets per litter	12.2		12.9		13.4		13.1		13.4		0.46	0.089	0.352
Litter weight at birth (kg)	17.8		18.3		18.8		18.1		19.4		0.51	0.117	0.846
Litter weight variation at birth (%)^2)^	22.7		24.2		23.9		25.2		21.4		1.31	0.664	0.066
Stillbirth per litter^3)^	1.58		1.30		0.90		0.94		0.90		0.23	0.017	0.286
Litter mortality rate at birth (%)	15.9		13.7		7.87		10.8		12.3		1.86	0.076	0.020
Total born piglets alive per litter	10.3		10.9		11.8		11.6		11.5		0.21	<0.001	0.003

SEM, standard error of the mean.

1) MG, mid-gestation (days 42 to 76); LG, late gestation (days 77 to 103).

2) Values for coefficient of variation.

3) Stillbirth contains mummies.

**Table 4:** Estimated standardized ileal digestible lysine requirement based on total born piglets alive per litter with four different statistical model^1)^

Item	Statistical model							

	One slope broken-line		Quadratic^2)^		Former intercept^3)^		Quadratic broken-line	
			
	MG	LG	MG	LG	MG	LG	MG	LG
Req. (g/d)	9.7	14.6	12.4	17.4	11.0	16.2	11.4	16.5
SE	1.08	1.16	0.91	0.86	-	-	1.88	2.18
p-value	<0.01	<0.01	<0.01	<0.01	-	-	<0.01	<0.01
Req. (g/Mcal ME)^4)^	1.19	1.62	1.52	1.93	1.35	1.80	1.40	1.83

SE, standard error.

1) MG, mid-gestation (days 42 to 76); LG, late gestation (days 77 to 103); Req., requirement.

2) 95% of the upper asymptotic value for MG and LG, 12.4 and 17.4 g/d.

3) Values for between the quadratic model and the plateau line of one slope broken-line.

4) Calculated values based on the average daily feed intake (2.5 and 2.8 kg in mid and late gestation) and metabolizable energy (ME) concentrations (3,265 and 3,221 kcal in mid and late gestation) in diets.

**Table 5:** The effects of standardized ileal digestible (SID) lysine (Lys) concentrations on lactation performance in sows^1)^

Item	Standardized ileal digestible lysine (%)										SEM	p-value	
	
	MG	LG	MG	LG	MG	LG	MG	LG	MG	LG		Linear	Quadratic
				
	0.22	0.36	0.32	0.46	0.42	0.56	0.52	0.66	0.62	0.76			
Average daily feed intake (kg/d)	2.50	2.80	2.50	2.79	2.51	2.78	2.51	2.80	2.47	2.77	-	-	-
SID Lys intake (g/d)	5.48	10.5	8.00	12.8	10.5	15.5	13.0	18.5	15.3	21.0	-	-	-
Number of lactating sow^2)^	37		37		33		37		38		-	-	-
Lactation period (d)	19.9		19.8		19.7		19.4		19.5		-	-	-
Number of fostering piglets per litter	10.2		10.9		11.8		11.6		11.5		0.21	<0.001	<0.001
Litter weight at fostering (kg)	16.2		15.8		17.5		17.1		17.7		0.50	<0.001	0.902
Litter weight gain (kg)	43.7		46.6		46.5		47.3		48.3		1.59	0.050	0.607
Litter weight at weaning (kg)	58.5		61.6		61.8		62.1		64.0		1.90	0.051	0.770
Litter weight variation at weaning (%)^3)^	16.7		18.4		17.9		18.3		18.0		0.98	0.415	0.403
Litter mortality rate at weaning (%)	7.96		7.78		15.4		14.0		12.6		2.18	0.023	0.163
Number of piglets at weaning per litter	9.05		10.0		9.87		9.86		10.0		0.25	0.026	0.114

SEM, standard error of the mean.

1) MG, mid-gestation (days 42 to 76); LG, late gestation (days 77 to 103).

2) After farrowing, the sows were excluded from observations in case of disability in lactating.

3) Values for coefficient of variation.

**Table 6:** The effects of standardized ileal digestible lysine concentrations on subsequent reproductive performance in sows^1)^

Items	Standardized ileal digestible lysine (%)										SEM	p-value

	MG	LG	MG	LG	MG	LG	MG	LG	MG	LG		
				
	0.22	0.36	0.32	0.46	0.42	0.56	0.52	0.66	0.62	0.76		
Number of sows	37		37		33		37		38		-	-
Average parity	5.00		5.08		5.00		5.11		5.08		-	-
Longevity (number of sows)												
Farrowed	33		32		27		35		35		-	-
Culled	0		3		4		1		3		-	-
Aborted	4		1		2		1		0		-	-
Disabled	0		1		0		0		0		-	-
Average parity	5.84		5.86		5.85		5.95		5.95		-	-
Production performance												
Total born piglets per litter	11.4		12.4		11.3		12.0		12.1		0.52	0.540
Number of piglets at weaning	8.73		9.95		9.97		9.51		9.63		0.32	0.047
Weaning to estrus interval (d)	4.21		7.10		8.37		5.13		7.40		1.28	0.138

SEM, standard error of the mean.

1) MG, mid-gestation (days 42 to 76); LG, late gestation (days 77 to 103).
